# Characteristics of post hoc subgroup analyses of oncology clinical trials: a systematic review

**DOI:** 10.1093/jncics/pkad100

**Published:** 2023-11-25

**Authors:** Jawad Alrawabdeh, Marah Alzu'bi, Muntaser Alzyoud, Nada Odeh, Yazan Hamadneh, Hira Mian, Ghulam Rehman Mohyuddin, Amar H Kelkar, Aaron M Goodman, Rajshekhar Chakraborty, David A Russler-Germain, Nikita Mehra, Diva Baggio, Edward R Scheffer Cliff, Samer Al Hadidi

**Affiliations:** School of Medicine, University of Jordan, Amman, Jordan; School of Medicine, University of Jordan, Amman, Jordan; School of Medicine, University of Jordan, Amman, Jordan; School of Medicine, University of Jordan, Amman, Jordan; School of Medicine, University of Jordan, Amman, Jordan; Department of Oncology, McMaster University, Hamilton, ON, Canada; University of Utah, Salt Lake City, UT, USA; Department of Medical Oncology, Dana-Farber Cancer Institute, Boston, MA, USA; Harvard Medical School, Boston, MA, USA; University of California San Diego, La Jolla, CA, USA; Herbert Irving Comprehensive Cancer Center, Columbia University Irving Medical Center, New York, NY, USA; Division of Oncology, Department of Medicine, Washington University School of Medicine, St. Louis, MO, USA; Department of Medical Oncology, Cancer Institute (WIA), Chennai, Tamil Nadu, India; Peter MacCallum Cancer Center, Parkville, VIC, Australia; Harvard Medical School, Boston, MA, USA; Program on Regulation, Therapeutics and Law, Division of Pharmacoepidemiology and Pharmacoeconomics, Brigham and Women’s Hospital, Harvard Medical School, Boston, MA, USA; Myeloma Center, Winthrop P. Rockefeller Cancer Institute, University of Arkansas for Medical Sciences, Little Rock, AR, USA

## Abstract

**Background:**

Subgroup analyses in clinical trials assess intervention effects on specific patient subgroups, ensuring generalizability. However, they are usually only able to generate hypotheses rather than definitive conclusions. This study examined the prevalence and characteristics of post hoc subgroup analysis in oncology.

**Methods:**

We systematically reviewed published subgroup analyses from 2000 to 2022. We included articles presenting secondary, post hoc, or subgroup analyses of interventional clinical trials in oncology, cancer survivorship, or cancer screening, published separately from the original clinical trial publication. We collected cancer type, year of publication, where and how subgroup analyses were reported, and funding.

**Results:**

Out of 16 487 screened publications, 1612 studies were included, primarily subgroup analyses of treatment trials for solid tumors (82%). Medical writers contributed to 31% of articles, and 58% of articles reported conflicts of interest. Subgroup analyses increased significantly over time, with 695 published between 2019 and 2022, compared to 384 from 2000 to 2014. Gastrointestinal tumors (25%) and lymphoid lineage tumors (39%) were the most frequently studied solid and hematological malignancies, respectively. Industry funding and reporting of conflicts of interest increased over time. Subgroup analyses often neglected to indicate their secondary nature in the title. Most authors were from high-income countries, most commonly North America (45%).

**Conclusions:**

This study demonstrates the rapidly growing use of post hoc subgroup analysis of oncology clinical trials, revealing that the majority are supported by pharmaceutical companies, and they frequently fail to indicate their secondary nature in the title. Given the known methodological limitations of subgroup analyses, caution is recommended among authors, readers, and reviewers when conducting and interpreting these studies.

Subgroup analysis plays a crucial role in reporting the findings of randomized clinical trials (RCTs), and it is typically intended to evaluate whether the findings apply homogenously across included patients (1,2). However, these analyses, now often conducted post hoc rather than being prespecified, assess the impact of an intervention on outcomes within particular patient subgroups, typically identified by patients’ baseline characteristics (3).

Subgroup analysis may help to identify patient-specific factors that influence treatment effectiveness or toxicity, refine target demographics, and/or reduce unnecessary treatment. For example, utilization of a 21-gene assay in breast cancer has the potential to identify a substantial proportion (up to 85%) of women with early hormone receptor-positive, human epidermal growth factor receptor 2 (HER2)-negative breast cancer who may not require adjuvant chemotherapy (4). Subgroup analysis according to patients’ age found chemotherapy could be safely omitted for women over 50 years with a recurrence score less than or equal to 25 and women aged ≤50 years with a recurrence score ≤15 (4).

Subgroup analysis can sometimes identify patient subgroups who may benefit from interventions in negative trials—such as, potentially, venetoclax in translocation (11; 14) multiple myeloma (5)—which may prevent scientific losses, although these findings must typically be verified in subsequent prospective clinical trials (6,7). However, improper assessment or interpretation of subgroup analysis in clinical trials may result in unnecessary withholding of interventions or the implementation of ineffective or harmful treatments in clinical practice (2,8). Subgroup analyses must be undertaken with caution, as limitations of multiplicity testing and inadequacy of statistical power can reduce the informative value of subgroup analysis and increase the risk of false-positive or false-negative results (6,9-11).

For our study, subgroup analyses were defined as secondary analyses in articles separate from the original trial report. Previous preliminary assessments of subgroup analyses in oncology clinical trials are limited to specific malignancies, time periods, or phases of the original trials (6,12,13). Therefore, we sought to comprehensively investigate subgroup analyses of oncology clinical trials to determine their prevalence over time, describe the characteristics of these analyses, and explore the associations between their characteristics to better understand the appropriateness of their use and interpretation.

## Methods

### Search strategy

We conducted a comprehensive search with the Ovid MEDLINE database to identify published original studies describing secondary, post hoc, or subgroup analysis of oncology clinical trials. We defined subgroup analyses as secondary analyses of previously published clinical trials, published subsequently as separate articles (not part of the initial publication, which typically report on prespecified subgroup analyses). We emphasized “separate articles from the original,” as they are often post hoc explorations of subgroups not originally specified in the trial protocol. Our focus on post hoc subgroup analyses is motivated by their susceptibility to biases and increased risk of generating false-positive results. Conversely, prespecified subgroup analyses, integrated into the initial trial publication, are more likely to be intrinsic to the original study design, and many prespecified analyses are both reasonable and necessary to ensure the consistency of treatment effects across diverse groups. The following search term was used: “(exp Neoplasms/or Neoplasm* or tumor* or cancer* or lymphoma or myeloma or adenocarcinoma or carcinoid or sarcoma or leukemia or carcinoma or tumor or dyscrasia or Hodgkin’s or melanoma or neoplasia or glioblastoma or astrocytoma or medulloblastoma or myelodysplastic or craniopharyngioma or ependymoma or retinoblastoma or neuroblastoma or papillomatosis or thymoma or blastoma or rhabdomyosarcoma or pheochromocytoma) AND (subgroup analysis* or post-hoc analysis or post-hoc analysis or exploratory analysis or correlational analysis or post hoc analysis or interaction analysis or secondary analysis or posteriori analysis) NOT (systematic review or meta-analysis).tw.kw.”

Our search targeted titles, abstracts, and keywords, and we limited the results to articles published in English from January 1, 2000, to February 14, 2022. Our study adhered to the Preferred Reporting Items for Systematic Reviews and Meta-Analyses (PRISMA) guideline (14).

### Screening

Five authors (J.A., M.A., M.A., N.O., Y.H.) collaborated to determine the eligibility of studies for inclusion in the analysis, conducting regular meetings with the corresponding author (SAH). The initial screening phase involved the evaluation of 40 articles, which were reviewed by all five authors. Subsequently, a comparative analysis was performed to assess articles for inclusion or exclusion, aiming for a unified screening method. Articles were included for full review if there was any doubt. After this initial screening, a total of 1963 articles remained under consideration. Multiple deliberative meetings, involving the corresponding author (SAH), were convened to address uncertainties related to these articles. Furthermore, during the data extraction phase, all authors independently re-evaluated each article to ensure its appropriateness for inclusion.

The criteria for inclusion were articles presenting secondary, post hoc, or subgroup analysis of interventional clinical trials in the field of cancer, cancer survivorship, or cancer screening in a separate publication from the original publication of the trial data. Titles, abstracts, and keywords were used to determine inclusion. When unclear, the full text was reviewed.

**Figure 1.**
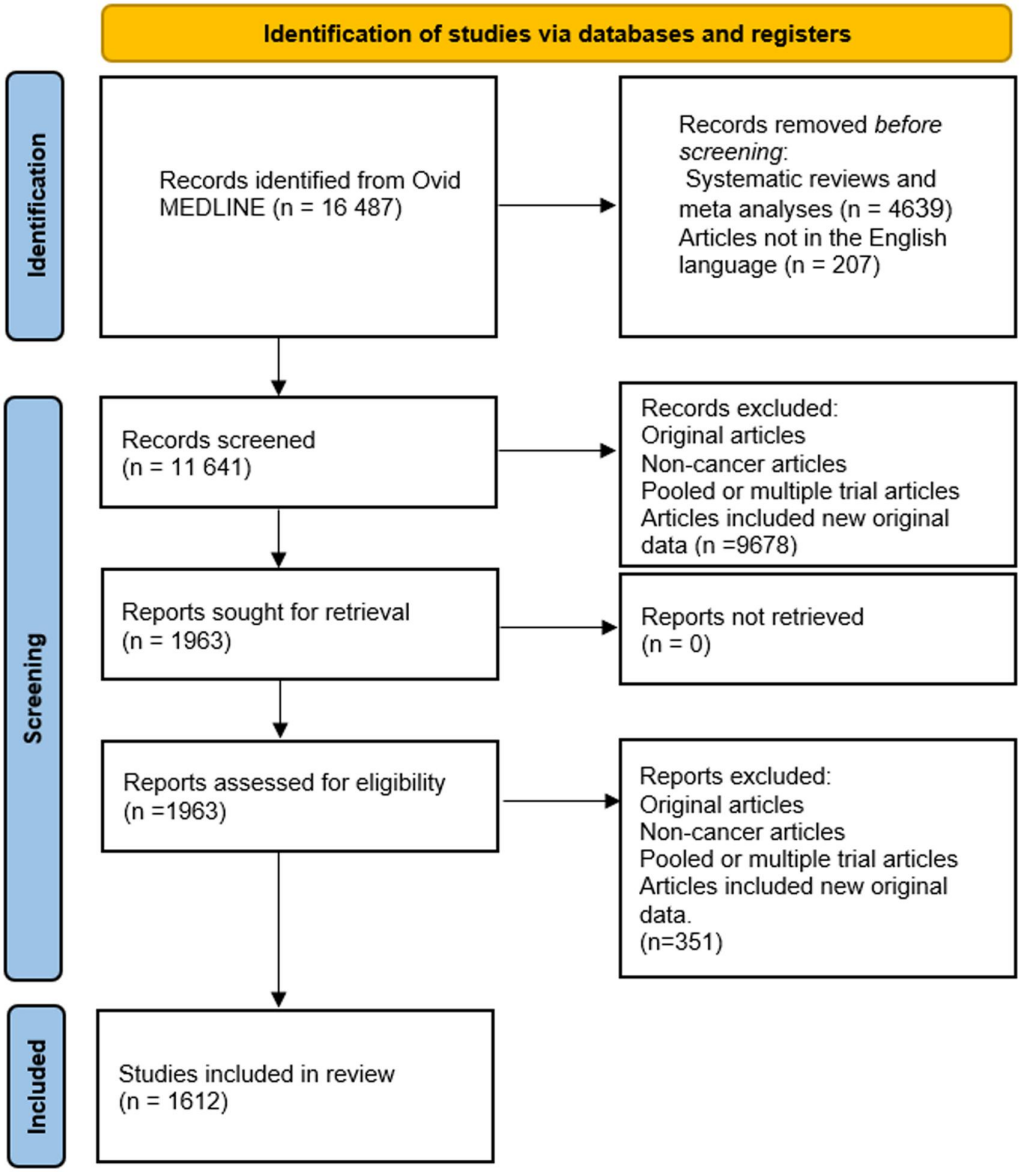
PRISMA 2020 flow diagram for the article screening and selection process.

After the screening, a total of 1612 articles were included for data extraction. The PRISMA Flow Diagram outlining the screening process is depicted in Figure 1 (14).

### Data extraction

We extracted the following variables for each article, where available: title, National Clinical Trial (NCT) number, country where the trial was conducted, type of trial (intervention on patients with cancer, either drug or nondrug; cancer survivorship; or cancer screening), type of cancer, year of publication, number of authors, journal name, reporting of the subgroup analysis in the article and in its title, funding status, medical writer assistance, and presence of conflicts of interest (COI). For articles published electronically before print, we used the electronic publication date as the year of publication. We defined anticancer drug trials expansively to include both directly anti-neoplastic medications such as chemotherapy as well as supportive medications (eg, antiemetics, growth factors, opioids). Non-drug interventional trials were defined as any intervention that is not a cancer or supportive care medication specifically (eg, behavioral therapy, exercise, psychosocial interventions). In the initial screening of most reviewed publications, there was a lack of explicit information concerning the pre-planning of subgroup analyses. During the initial data collection phase, we attempted to gather information indicating whether subgroup analyses had been pre-planned. However, due to the rarity of pre-planned analyses in the data set, we decided not to pursue further data collection in this regard.

Country information for the trials was obtained from the Methods section or NCT website, with unavailable data marked as not available (N/A). Trials were categorized as “survivors” for cancer survivors; “screening” for cancer screening; and “intervention” for medical interventions such as chemotherapy, surgery, radiation, or non-drug interventions. Cancer types were classified as solid or hematological, followed by specific organ systems. COIs were coded as “yes” if reported by any author and “no” if not reported by any author or unavailable, with the COI percentage calculated for each article separately based on authors reporting conflicts divided by the total number of authors for that article.

Author countries were categorized into regions and income levels (high-income countries and/or middle-/low-income countries) based on World Bank classifications, with regions including East Asia and Pacific, Latin America and the Caribbean, Europe and Central Asia, Middle East and North Africa, North America, South Asia, and sub-Saharan Africa.

We assessed the reporting of subgroup analysis in article titles, abstracts, and Methods sections, considering explicit mentions of “post hoc,” “secondary,” or “subgroup analysis.” Articles were categorized into 8 reporting status categories based on where in the article the nature of the analysis was reported, including: not explicitly stated in any section, title only, abstract only, methods only, title and abstract, title and methods, abstract and methods, or stated in all sections. A simple measure was developed to facilitate comparisons such that an article received a point for explicitly stating the status in the title, abstract, and/or Methods sections for a maximum of 3 points. Articles that did not have a Methods section were considered “missing.”

### Data analysis

Descriptive statistics were used to analyze the data. Chi-square tests were used to assess associations between categorical variables. Phi and Cramer’s V were used as effect size measures, and adjusted standardized residual values were used for post hoc testing. Fisher’s exact test was used when the chi-square assumption was violated. *T*-tests and Analysis of Variances were used to find differences in means between variables. A time series analysis was used to predict future subgroup analyses. Data analysis was conducted to investigate the presence of autocorrelation in the time series data. The Ljung-Box Q-test was applied to assess the statistical significance of correlations between consecutive data points. This test is commonly employed in time series analysis to examine the presence of autocorrelation, which is when a data point is related to previous data points in the sequence. An Auto-Regressive Integrated Moving Average Model (ARIMA) was used with (p, d, q) values of (0,1,0).

## Results

### Characteristics of the articles

In total, 1612 articles were included for analysis. The majority (77%) of these articles were subgroup analyses of interventional trials of anticancer drugs, followed by non-drug interventional trials (16%), screening trials (3.7%), and cancer survivorship trials (3.5%) (Table 1). A total of 82% focused solely on solid tumors, and the remaining articles explored hematological malignancies or both. In terms of location, 20% of trials took place exclusively in the United States, 4% in Japan, 16% in other single countries, and 40% involving multiple countries. In ∼10% of articles, trial location was not specified. About 54% (n = 827) of all articles included US patients, and 69% (1113) included patients from non-US countries.

**Table 1.** General characteristics of subgroup analyses (n = 1612)

Variable	Frequency	Percentage
Type of trial		
Intervention-drug	1236	76.7
Intervention-nondrug	259	16.1
Survivors	57	3.5
Screening	60	3.7
Cancer type		
Solid	1322	82.0
Hematological	160	9.9
Both	130	8.1
Year of publication		
2000-2014	384	23.8
2014-2018	533	33.1
2018-2022	695	43.1
Country of patients		
US	326	20.2
UK	33	2.0
Germany	64	4.0
Japan	68	4.2
Canada	51	3.2
Other	265	16.4
More than one	640	39.7
Not available	165	10.2
Did the trial include: US patients	827	54.1
Did the trial report funding? (Missing n = 1)		
Yes	1288	80.0
No	323	20.0
Did the trial report pharmaceutical company funding? (Missing n = 1)		
Yes	731	45.4
No	880	54.6
Medical writer used? (Missing n = 1)		
No	506	68.6
Yes	1105	31.4
Conflicts of interest reported?		
Yes	937	58.1
No	675	41.9

### Time trend of subgroup analyses

**Figure 2.**
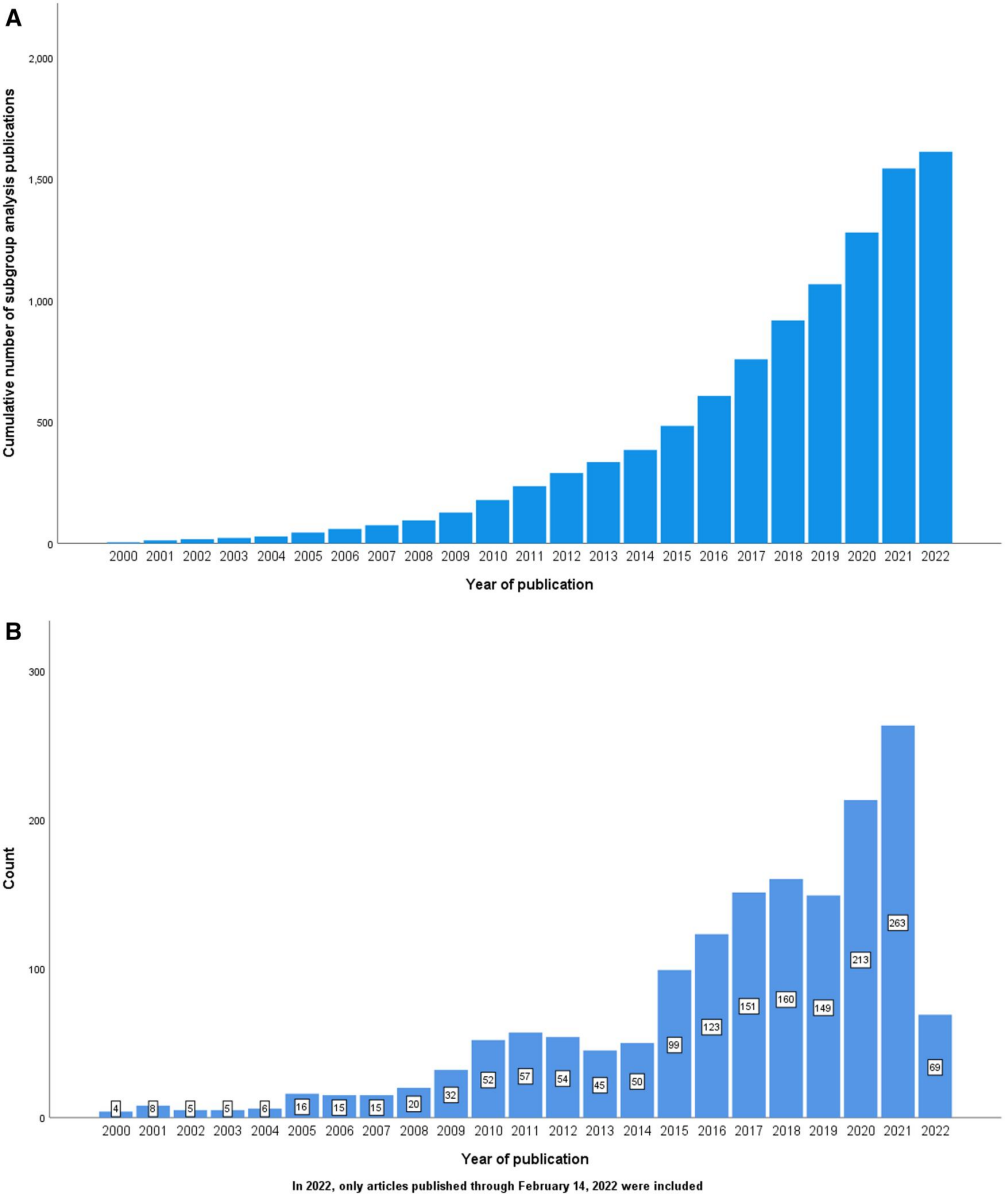
(**A**) Cumulative frequency of subgroup analysis according to publication year. (**B**) Number of subgroup analyses per year.

The number of published subgroup analyses increased over time. In 2000, only 4 subgroup analyses were published; in 2010, 52 were published, and in 2021, 263 subgroup analyses were published (Figure 2). To facilitate comparison, we categorized the years of publication into 3 periods: 2000-2014, 2015-2018, and 2019-2022. During the substantially longer first period, there were 384 articles (24%); the second period had 533 articles (33%), and the last period had 695 articles (43%).

To forecast the future number of subgroup analyses, we used an ARIMA time series model, which suggested that there are likely to be 275 articles in 2022, 288 in 2023, 300 in 2024, 312 in 2025, and 324 in 2026. The model achieved an R-squared value of 0.927, with a mean absolute error of 15.333, indicating that our model fits the observed data relatively well. The Ljung-Box Q-test yielded a nonsignificant result (*P* = .213), suggesting no autocorrelation and therefore independent values.

### Most studied malignancies

Gastrointestinal tumors were the most common solid malignancies with reported subgroup analysis, accounting for 25.3% of cases (Table 2). Genitourinary cancers constituted 23.6% of the studied solid tumors, followed by breast cancer (21.6%). Among hematological malignancies, tumors of the lymphoid lineage were the most frequently studied, comprising 39.4% of hematological malignancies. Multiple myeloma ranked as the second most studied hematological malignancy with 35.6%, and myeloid lineage tumors accounted for most of the remainder (20%).

**Table 2.** Frequencies of studied solid and hematological malignances

Cancer type	Specific cancer	Frequency (percentage)
Solid tumors	Gastrointestinal	334 (25.3%)
	Genitourinary	312 (23.6%)
	Breast	285 (21.6%)
	Central nervous system	35 (2.6%)
	Endocrine	12 (0.9%)
	Head and neck	53 (4.0%)
	Respiratory	182 (13.8%)
	Musculoskeletal or sarcoma	11 (0.8%)
	Skin	39 (3.0%)
	More than one	40 (3.0%)
	Others	19 (1.4%)
Hematological malignancies	Lymphoid lineage	63 (39.4%)
	Multiple myeloma	57 (35.6%)
	Myeloid lineage	32 (20.0%)
	More than one	8 (5.0%)
Solid and hematological malignancies	More than one	130 (8.1%)

### Funding, medical writers, and conflicts of interest

**Figure 3.**
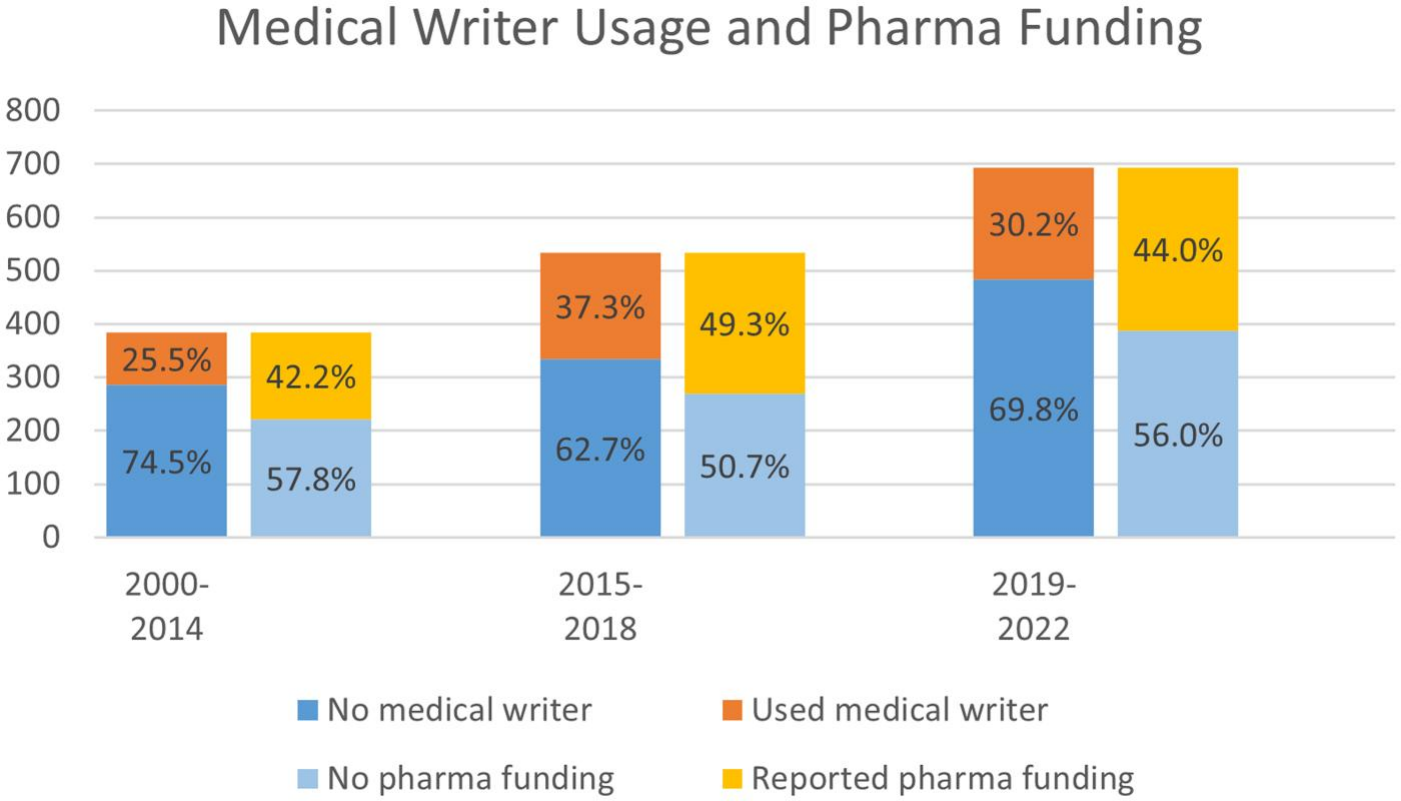
Usage of medical writing and pharmaceutical industry funding in subgroup analysis.

Medical writers were involved in the preparation of 31% of the articles, and COI was reported in 58%. The remaining 44% either did not report COI or reported no COI. The most recent period, 2019-2022, had a considerably higher proportion of articles reporting any kind of funding compared to other periods (*P* < .001). However, there was no significant change in the rate of funding from pharmaceutical companies over the years (*P* = .065), in the context of a far greater number of published subgroup analyses. The reporting of medical writer usage varied significantly between time periods, increasing from 25% from 2000 to 2014 to 37% from 2015 to 2018, and then down to 30% from 2019 to 2022 (*P* < .001, Cramer’s V = 0.1) (Figure 3). The reporting of COI increased significantly across all time periods, with the more recent periods having the highest rates of reporting (*P* < .001). Both the 2014-2018 and 2019-2022 periods exhibited significantly higher average percentages (approximately 40% for both) of COI compared to the earliest period (30%) (*P* < .001).

Interventional drug trials reported the highest percentages of funding, pharmaceutical company funding, COIs, and usage of medical writers compared to other types of trials (*P* < .001 for all). Subgroup analyses of hematological malignancies compared to solid and mixed cancer trials, respectively, showed higher percentages of studies that reported funding (89.4% vs 78.1% vs 86.9%), pharmaceutical company funding (68.8% vs 44.8% vs 22.3%), reporting of COIs (80.9% vs 58% vs 35.4%), and usage of medical writers (50% vs 30.7% vs 16.2%) compared to solid and mixed cancer trials (*P* < .001 for all.).

### Reporting the secondary subgroup analysis status

We categorized the articles into 8 reporting categories based on where they reported the secondary subgroup nature of their analysis, if at all. Most commonly, 29% of articles reported the nature of the analysis in both the abstract and Methods sections (Table 3). Approximately one-quarter of the articles explicitly stated the subgroup analysis status in all sections. Around 10% of articles mentioned it solely in the title section. Around 50% of all articles did not include the subgroup analysis status in the title. A small portion of articles (3.2%) did not explicitly state it in any section.

**Table 3.** Frequencies of the reporting status of subgroup analysis usage in title, abstract, and methods

Method of assessing status	Reporting status	Count	Percentage
8 categories	Not in title, abstract, or methods	51	3.2%
	Only title	162	10.0%
	Only abstract	221	13.7%
	Title and abstract	132	8.2%
	Only methods	43	2.7%
	Title and methods	94	5.8%
	Abstract and methods	472	29.3%
	Title and abstract and methods	413	25.6%
	Missing	24	1.5%
Simple measure^a^	0	51	3.2%
	1	426	26.4%
	2	698	43.3%
	3	413	25.6%
	Missing	24	1.5%

a A point system developed to facilitate comparisons such that a publication received a point for explicitly stating the status in the title, abstract, and/or Methods section for a maximum of 3 points. Publications that did not have a Methods section were considered “missing.”

With the simplified points score described in the methods, the most frequent score was 2, representing 43% of articles. The reporting status significantly varied across different time periods: articles published in the 2019-2022 period were more likely to receive a score of 3, while being less likely to score 0 or 1 (*P* < .001). Furthermore, articles that reported the use of a medical writer, pharmaceutical company funding, and COI were more likely to receive a score of 3 (*P* < .001 for all 3). Trials funded by pharmaceutical companies were the most likely to use medical writers (*P* < .001).

### Regional representation in authorships

Regarding regional representation, more than 97% of first author, last author, and corresponding author institutional affiliations were based in high-income countries. Approximately 45% were from North America, followed by 34% from Europe and Central Asia, and approximately 20% from East Asia and the Pacific.

## Discussion

Our investigation revealed a substantial increase in the number of oncology subgroup analyses published over time: whereas 25% were published in the 14 years from 2000 and 2014, 45% were published in just the last 4 years. Around 50% of the studies in our analysis were funded by pharmaceutical companies, consistent with the previous literature (5,6); approximately 31% involved medical writers, and about 58% of coauthors reported COIs. Although time trends were mixed, the proportion of these subgroup analyses with pharmaceutical company and with medical writer involvement remains substantial, which, given their often post hoc nature, raises concerns of possible bias in these articles. This finding reflects the fact that most (98%) clinical trials that report medical writing support are funded by pharmaceutical companies (15).

It is essential that subgroup analyses clearly report the nature of their analysis to enable readers to appropriately interpret their typically provisional results. Our finding that 85% of these analyses did not report the secondary nature of their analysis in the title suggests that medical journals should have stricter rules to compel authors to include this information more clearly in article titles. Our findings regarding the proportion of articles that mention the nature of their analysis was similar to that of prior studies indicated that subgroup analysis status was mentioned in the abstract for at least 15% of the articles and in the Methods section in at least 40% (6,9-11).

Our study’s strengths include its breadth, longer time period, sample size, and inclusion of both solid and hematological malignancies.

The United States Food and Drug Administration guidance emphasizes the need for prespecification of subgroups, including gender, age, and race, and lays out the appropriate statistical approaches to assessing groups such as molecularly defined disease subsets and older adults (7). The United States Food and Drug Administration further highlights that subgroup analyses “must be put in the context of the magnitude of the drug’s effect on the subpopulation, the degree of the subgroup’s representation in the overall intention-to-treat population, and the biologic plausibility of a differential effect in the subgroup, among other factors.” Early attention should be given to patient subgroups with expected differences in outcomes, and comprehensive plans for patient accrual and statistical analysis should be implemented to adequately characterize these potential differences (16).

Subgroup analyses can be misleading if they aim to identify patient subpopulations who appear to benefit from an intervention despite a trial’s overall negative result. Positive outcomes in a subset of a failed experiment should not support the adoption of an intervention given that trials are typically not sufficiently powered to answer this question (ie, the sample size of the subgroup is often too small to enable robust statistical analysis) and should instead be tested in subsequent prospective trials (6,17). However, many researchers still incorporate subgroup analysis results in their study presentations, which can influence the interpretation of the overall findings (18).

Another concern in subgroup analysis is the issue of multiple hypothesis testing (19). When multiple subgroups are evaluated for treatment effects without appropriate adjustments, there is an increased likelihood of obtaining false-positive results by chance. If these analyses are not specified before the trial, it is hard to know how many analyses were conducted to generate the results that are presented or published.

Categorizing subgroup analyses as inferential or exploratory can be helpful to overcome these challenges. Inferential subgroup analysis is prespecified in the study protocol with careful planning, including sufficient sample size and control of alpha (significance) level.

Study limitations included that selective reporting bias may have influenced the number of subgroups investigated and reported in the published articles, as we relied solely on published reports and study protocols. We did not determine whether the original RCTs associated with the subgroup analyses were positive or negative, nor the proportion that were prespecified before the trial opening. Our analysis was limited to fully published articles.

We demonstrate that the use of subgroup analyses of oncology clinical trials is rapidly increasing, often supported by pharmaceutical companies. As our understanding of the pathogenesis of cancer subtypes improves and molecularly targeted therapies proliferate, treatments with differential effects in subgroups of patients are likely to become more common, highlighting that statistically robust approaches to subgroup analysis and nuanced communication of their findings are critical. Our findings, together with our knowledge of the methodological limitations of subgroup analyses, emphasize the need for authors, readers, journal editors, and reviewers to exercise caution in conducting and interpreting these studies.

## Data Availability

Data will be made available per request to the corresponding author for 1 year after publication.
